# Identifying gaps in protection from malaria vector biting in rural Cambodia using an entomological assessment and human behaviour observations

**DOI:** 10.1186/s12936-025-05304-x

**Published:** 2025-03-24

**Authors:** David J. McIver, Elodie A. Vajda, Dyna Doum, Nicholas W. Daniel, Molly Quan, Diane D. Lovin, Joanne M. Cunningham, Siv Sovannaroth, Allison Tatarsky, Neil F. Lobo

**Affiliations:** 1 Malaria Elimination Initiative, Institute for Global Health Sciences, University of California, 550 16th Street, San Francisco, CA 94158 USA; 2 Swiss Tropical and Public Health Institute, Socinstrasse 57, P. O. Box, CH-4002 Basel, Switzerland; 3 University of Basel, Petersplatz 1, CH-2003 Basel, Switzerland; 4 Health Forefront Organization, Phnom Penh, Cambodia; 5 University of Notre Dame, Notre Dame, IN 46556 USA; 6 National Center for Parasitology, Entomology and Malaria Control, 477 Betong St, Phnom Penh, Cambodia

**Keywords:** Malaria, Gaps in protection, Human landing catch, Human behaviour observation, Elimination, Topical repellent, Spatial repellent, Spatial emanator

## Abstract

**Background:**

Forest-exposed populations remain the last significant, and most difficult to access, high-risk populations for malaria in Cambodia. Despite the availability of long-lasting insecticidal nets (LLINs) and/or hammock nets (LLIHNs), continued malaria transmission indicates gaps in protection. This study aimed to identify these gaps among forest-exposed individuals in *Plasmodium falciparum* hotspots in two provinces in Cambodia, using entomological assessments and human behaviour observations (HBOs).

**Methods:**

*Anopheles* bionomic traits were characterized using Human Landing Catches (HLCs) in a village setting in Mondulkiri province, and in both village and forest settings in Kampong Speu province, Cambodia. Mosquitoes were collected from 17h00 to 07h00 over 540 collection nights. Human behaviour observations (HBOs) focused on monitoring activities near HLC sites and recording the use of LLINs/LLIHNs or Project BITE’s bite prevention tools: a volatile pyrethroid spatial repellent (VPSR), topical repellent (TR), and insecticide-treated clothing (ITC). Data on mosquito landing pressure and human behaviours were integrated to generate the HBO-adjusted Human Landing Rate (HBO-adjusted HLR).

**Results:**

A total of 5,985 *Anopheles* mosquitoes were collected, with 608 (10%) identified molecularly to species-level. Seventeen *Anopheles* species were identified, including a likely novel species from the Leucosphyrus Subgroup, which was the predominant species characterized. The HBO-adjusted HLR was found to be greatest during the early evening hours, when people were outdoors awake, followed by when people were sleeping indoors without a net. Relatively few people were observed using, or correctly using, the new bite prevention tools intended for protection in the forest.

**Conclusion:**

This study demonstrates the importance of understanding spatial and temporal human exposure to mosquito bites, in the presence of proven vector control tools (LLINs, LLIHNs) and newly introduced bite prevention tools (VPSRs, ITCs, and TRs). To help achieve malaria elimination, human behaviour data on intervention use and behaviour patterns should be evaluated and integrated with entomological data towards identifying and quantifying protection conferred by current interventions, as well as remaining gaps in protection. This information supports the selection of appropriate interventions, which supplement rather than replace existing tools, to target existing gaps in protection.

## Background

Cambodia is in the last mile of malaria elimination [1] and aims to eliminate all species of human malaria by 2025 [2]. As in other countries in the Greater Mekong Subregion, remaining areas of transmission in Cambodia are in forest settings [3]. From 2010 to 2022, confirmed malaria cases in Cambodia fell by 91%, with the disease becoming more confined to specific transmission foci. About 500,000 people in Cambodia live in forested and forest fringe areas with high malaria transmission [4–7], primarily affecting ethnic minorities, local populations, and rural mobile and migrant workers in rubber plantations, mining, and agriculture [8, 9]. Under the Malaria Elimination Action Framework (2021–2025), Cambodia's National Center for Parasitology, Entomology, and Malaria Control (CNM) distributes forest packs containing insecticide-treated nets (ITNs), insecticide-treated hammock nets, and topical repellents to mobile and migrant populations in high-risk areas [10–12]. Since November 2020, Cambodia has also implemented innovative foci-based strategies, including targeted drug administration and intermittent preventive treatment for forest-goers, as part of its "last mile" approach towards *Plasmodium falciparum* malaria elimination [11, 13].

However, with 1,384 cases reported in Cambodia in 2023 [14], effective mosquito bite prevention interventions are needed based on the specific places and times forest-going and forest-dwelling populations are exposed to *Anopheles* mosquitoes [5] to support the final push to elimination. A study in northern Cambodia observed that while *Anopheles* densities significantly decreased in villages during the dry season, they remained relatively stable in the forest, suggesting that forests may serve as refuge for *Anopheles* during the dry season and consequently, as reservoirs for malaria parasites [15]. Forest-goers face exposure to vector bites during both the day and night due to the outdoor, daytime, and early evening biting behaviour of A*nopheles* [16], along with low bed net use and open sleeping structures [15, 17], thereby reducing the effectiveness of traditional village- and homestead-centric vector control methods. To introduce relevant new tools in the most efficient and effective way possible, it is important to understand where existing tools are *not* providing protection and pair that information with evidence on how additional or alternative tools could address those gaps.

Human behaviour observations (HBO) offer one method to determine drivers of exposure by characterizing how people spend their time, including where (indoors or outdoors), and when [18]. These data can be combined with evidence on local vector behaviour (either through existing information or by conducting parallel mosquito collection studies, such as human landing catches (HLCs)), to determine where human activities overlap with vector activities. Taken one step further, when the use of vector control tools by the study population is also considered during the HBO activities, a “map” can be created which reveals and quantifies where and when people *are* protected by the existing vector control tools, and where and when they are *not* protected (i.e., *gaps* in protection) [19]. As an example, an individual may have access to a high-quality and intact LLIN in their home, and sleeps under it at night. However, if the local vector population exhibits indoor biting during the early evening hours, the LLIN offers no protection to the individual spending time indoors before going to sleep under the protection of the net. By identifying the times and places where people are unprotected against malaria vectors by existing interventions, and by understanding their activity profiles during these times, the appropriate, complimentary interventions can be selected and targeted alongside the core interventions to further reduce human-vector exposure.

Novel vector control tools are continuously being developed and studied. Project BITE (Bite Interruption Toward Elimination) (2020–2023), a multi-stage research programme, aimed to investigate the efficacy, functionality, acceptability, and feasibility of novel, mosquito bite prevention interventions for forest-exposed populations. Project BITE evaluated volatile pyrethroid spatial repellents (VPSRs), insecticide treated clothing (ITCs), and a topical repellent (TR) in both the semi-field system in Thailand [20] and in the field in Cambodia [21]. In addition, Project BITE also collected acceptability data from forest-exposed individuals who used the tools in real-life situations [22]. VPSRs use pyrethroids that work in the vapor phase (for example, transfluthrin or metofluthrin) and reduce human-vector exposure through *non-contact* irritancy, non-contact excitorepellency, spatial repellency, landing inhibition, feeding inhibition, sublethal incapacitation, and mortality [23, 24]. ITCs offers another approach to addressing gaps in protection, since once the clothing is treated, users can benefit from its bite protection when stationary or moving, and in any setting (including at home, at work, in the forest) [21, 25, 26]. ITCs reduce human-vector exposure through contact irritancy, contact excitorepellency, some short-range non-contact excitorepellency, feeding inhibition, and mortality [25–27]. Synthetic topical repellents like picaridin and DEET provide personal protection against mosquito bites via short-range processes, such as blocking olfactory attractants [28, 29], inducing non-contact irritancy, and/or direct contact irritancy [30, 31]. Pairing ITCs with a topical repellent may further enhance bite prevention [32].

As part of Project BITE’s large-scale implementation feasibility study, forest packs containing a transfluthrin-based VPSR, an etofenprox solution for treating clothing, and a topical repellent, were delivered to 5,744 forest-exposed people in Cambodia. Recipients of the forest packs were encouraged to use the tools as much as possible and especially in the forest, and in combination when practical, toward decreasing their risk of *Anopheles* bites. Since these tools were all new to the study population, social and behaviour change communication (SBCC) strategies were also employed alongside the forest pack, to encourage appropriate and correct use of each tool.

The primary objective of the study described in this paper was to identify and quantify the gaps in protection among forest-exposed populations in *P. falciparum* hotspots in Mondulkiri and Kampong Speu provinces, Cambodia, using integrated entomological assessments and HBOs across the same time period and sites of the large-scale implementation feasibility study. This sub-study’s secondary objectives were to (1) describe the *Anopheles* species diversity in the village (Mondulkiri, Kampong Speu) and forest (Kampong Speu) settings; and (2) observe using HBOs, the population’s use of Project BITE forest pack tools to provide insights into how these novel interventions might help close the observed gaps in protection with the currently implemented tools.

## Methods

Entomological and human behavioural data for this field study were collected at three different timepoints. Timepoint 0 (T0) occurred between October 7–25, 2022, before the BITE forest packs were distributed. Following distribution of BITE forest packs, T1 occurred between December 3–18, 2022, and T2 between January 25–February 8, 2023. Field activities took place in three study sites: one in Mondulkiri and two in Kampong Speu. Study sites were selected due to their ongoing participation in the larger Project BITE programme (and thus presence of forest-exposed at-risk populations), high *P. falciparum* incidence (for the country), and their proximity to forested areas.

### Study sites and entomological collections (human landing catches, HLCs)

In Mondulkiri, all collections took place in a village setting located 1–2 km from the forest, Andoung Kroleong village (12.320725, 107.029779). Collections were carried out in eight different sentinel sleeping structures. In Kampong Speu, collections took place in both Peam Lvea village (11.407108, 104.068908), in six sentinel sleeping structures, and in a forest setting (11.401395, 104.064408) in two sentinel sleeping structures. Sentinel sleeping structures were selected such that each were at least 20 m away from each other, and the more open structure types (constructed out of thatch) were prioritized to reflect the open structures used in the forest. The Kampong Speu village sentinel structures were further from the forest compared to those in Mondulkiri, and the forest sentinel structures were in the forest fringe near a well-used pathway to enter the forest. At the forest sampling site, two temporary sleeping structures similar to those observed in the field were erected by the study team for conducting the HLCs, but there was no paired living structure at these locations (i.e., only outdoor collections were conducted, no indoor collections). An overview of the entomological sampling framework is provided in Table 1.

**Table 1:** Human Landing Catch (HLC) sampling overview

	**Mondulkiri**	**Kampong Speu**
Village sentinel sleeping structures (indoor/outdoor collections)	8	6
Forest sentinel sleeping structures (outdoor collections)	0	2
Number of collection nights per timepoint (6 nights per site)		
Indoor	48	36
Outdoor	48	48
Three timepoints (T0, T1, T2)	× 3	× 3
Total collection-nights	288	252

Paired HLCs were performed inside and outside sentinel sleeping structures (outside only for the two Kampong Speu forest sentinel structures) using 24-h collections for T0, and 14-h collections for T1 and T2, as the number of mosquitoes caught between 06h00 and 18h00 were minimal at T0 and, therefore, timeframes for subsequent collections were reduced.

The HLC collector outside each selected village structure was positioned within 3 m from the assigned structure. All HLC collectors sat on a chair and captured all mosquitoes landing on the exposed lower legs (exposed knee to exposed feet) using an aspirator, while wearing a head torch. Mosquitoes were collected in a catch cup covered with white, untreated netting, labelled with collector ID, structure ID, and collection hour. During every HLC collection hour, mosquito collections were conducted for 45 min, allowing for 2–3 min for the HBOs (more information below) and the remaining ~ 10 min for a rest and refreshment break. One supervisor assisted the collectors, verifying that the protocols were followed and ensuring the safe delivery of collected mosquitoes to the field laboratory. At T0, for each structure involved in the HLC collections, four collectors worked in 6-h shifts, rendering a total of 24 h of HLC collections per structure, per site. Based on results from the 24-h T0 HLC collections, indicating minimal mosquito activity between the hours of 06h00-17h00, collection hours were reduced from 17h00-07h00 for T1 and T2 collections.

HLC collectors were recruited from the local village and well known within the community. All collectors were men of 18 years or older, spoke Khmer or Bunong, and all provided informed consent to partake in the collections. Collectors were not permitted to drink, smoke, eat (except during their assigned hourly breaks) or wear perfume (or any other highly fragrant product such as strong scented deodorant). In accordance with local government guidelines, all mosquito collectors and field supervisors were tested for malaria before the start of the study, and after their last HLC collection at a site, and 14 days after their last collection, using nationally recommended RDTs. No positive diagnoses were recorded. Malaria prophylaxis was not prescribed to the collectors during the HLC collections because malaria prophylaxis is not required as per national recommendations. For all households that were included in indoor behaviour observations, informed, verbal consent was received to observe their actions during days and evenings.

### Human behaviour observations (HBOs)

In each sentinel sampling site, HLC collectors used the HBO method [18] to document intervention use and sleeping patterns, both indoors and outdoors, at the same structure they were collecting the entomological data. Study staff, recruited from the local village and well known within the community, observed study participants’ location, sleeping behaviour, and interventions being used, for each hour of the entomological collections. For all HLC collection hours, at 45 min passed each HLC collection hour, the collector paused to record the following information, indoors and outdoors: 1) number of people asleep, with a bed net; 2) number of people asleep, without a bed net; 3) number of people awake, without a bed net.

Additionally, for timepoints T1 and T2, the number of VPSRs hanging was also counted (counted the number of sheets). During timepoints T1 and T2, the HLC collectors also recorded etofenprox-treated clothing and topical repellent use by asking individuals around them if they were wearing etofenprox-treated clothing, and if they had applied the 20% picaridin topical repellent in the last hour, for HLC collection hours outside sleeping hours (21h00 – 06h00). For outdoor observations, the HLC collector only considered individuals within a 10-m radius of the sentinel sampling structure. Note that for the Kampong Speu forest site, the HLC collectors did not make any observations about the use of BiteBarriers, since these two temporary, sentinel sleeping structures were constructed by the study team for this field study. However, HBOs were conducted to record any observed outdoor LLIN/LLHIN use or non-use, sleep and awake patterns, topical repellent application, and wearing of etofenprox-treated clothing for any community members the collector will have observed as being within the vicinity of the temporary structure. Observations took place each hour and documented the following information: number of people indoors and outdoors (within 3 m of study structure), number of people using non-BITE vector control tools, and number of people using BITE tools (timepoints T1 and T2 only).

### Project BITE bite prevention tools

All households that took part in the larger implementation feasibility study were provided with a collection of bite prevention tools in the form of a “forest pack” which contained: 1) a 20% picaridin (191 g/L) topical repellent (OFF! Tropical Strength Insect Repellent Spray, SC Johnson & Son Pty Ltd), 2) a passive, transfluthrin-based VPSR (BiteBarrier, formerly known as PIRK, PIC Corp), and 3) a liquid etofenprox solution (Perimeter ETO Insect Guard formulation) for treatment of clothing (Pine Belt Processing Inc., a wholly owned subsidiary of Warmkraft, Inc.). The topical repellant requires active use (spraying on the user’s exposed skin) while the BiteBarrier is a passive product that can be hung up and left active. More information about these tools can be found at Vajda et al*.* [21]. While the purpose of providing these tools to at-risk communities was not to measure the impact of the tools on mosquito landing or behaviour (previous studies by the Project BITE research team conducted that work here: [21]) households were encouraged to use the products as much as possible, in combination, and both at home and when travelling to the forest, to protect themselves from mosquito bites. Distribution of BITE forest packs took place after the initial data collection activities at T0, and therefore human behaviour related to forest pack tool use is reported only for T1 and T2.

### Sample processing and molecular identification of mosquitoes

After completion of all nightly HLC collection activities, collection cups with mosquitoes were transported back to a field station. There, all mosquitoes were killed by freezing, and were subsequently sorted to genus-level [33]. All non-*Anopheles* mosquitoes were counted and then discarded. Mosquitoes were sorted morphologically to species-level or to the next highest taxon using a stereomicroscope and identification keys from Thailand [33]. The morphologically identified specimens were stored in Eppendorf tubes with desiccant (silica gel), and stored away from the humidity, heat, and sunlight. These samples were shipped to the University of Notre Dame, Indiana, USA, for subsequent molecular analysis.

A random sample of approximately 12% (n = 420 of 3,546 *Anopheles* collected across all three collection timepoints), 11% (n = 211 of 1,964), and 4% (n = 19 of 474) of each morphologically identified species from Mondulkiri, Kampong Speu village, and Kampong Speu forest, respectively, were randomly selected and sequenced at the ribosomal DNA internal transcribed spacer region 2 (ITS2) and/or cytochrome oxidase subunit 1 (CO1) loci towards species determination [17]. Conservative molecular species identification was based on matches to GenBank (National Center for Biotechnology Information [NCBI]) and BOLD [34] (databases with lower quality matches and an absence of voucher specimens resulted in identifications to higher taxonomic levels).

Human landing rate (HLR) and HBO-adjusted human landing rate (HBO-adjusted HLR).

The human landing rate (HLR) was calculated by determining the number of *Anopheles* mosquito landings per individual, per site (indoors/outdoors), per hour (lph) over the course of one night. To incorporate the HLR data with the HBO data, an HBO-adjusted HLR for each observed activity was calculated following the method outlined in Monroe et al*.* [18, 35, 36]. The adjusted human landing rate (HBO-adjusted HLR) is the HLR for a select number of different activities participants are observed doing, using data collected from HBOs. Observed activities contributing to HBO-adjusted HLR include: inside or outside, awake or asleep, under a bed net or not under a bed net. The HBO-adjusted HLR is used to quantify protection from mosquitos and elucidate where gaps in protection exist and is calculated using the total number of mosquitoes captured, both indoors and outdoors, for each HLC hour, and the proportion of people observed for each HBO indicator measured, during all HLC hours. The HBO-adjusted HLR represents the number of potential mosquito bites per hour, for a site, based on the observed HLR and protective behaviours taken by participants, and is expressed as the following equation:

$$HBO-adjusted HLR ={HLR}_{i}\times {proportion activity}_{lan}$$

where *i* represents the mean HLR at HLC collection hour *i*, *proportion activity*_*l*_ represents the individual’s location of the activity (indoors or outdoors), *proportion activity*_*a*_ represents the individuals conscious state (awake or asleep), and *proportion activity*_*n*_ represents the individual’s use of a mosquito net (net usage or no usage). There is a total of six combinations of *proportion activity*_*lan*_ (example: indoors, awake, no net).

## Results

### Molecular *Anopheles* species confirmation of specimens from HLC collections

A total of 17 *Anopheles* species were confirmed molecularly across both sites and all three time points (Table 2). Species and densities were related to each site and timepoint. The six species documented at Mondulkiri included a Subgroup Leucosphyrus species (an unknown species comprising 42% of all *Anopheles* captured in Mondulkiri), *Anopheles dirus A, An. dirus *sensu lato (*s.l*.), *Anopheles maculatus, Anopheles karwari,* and *Anopheles sawadwongporni*. The 15 species seen in Kampong Speu included the unknown Subgroup Leucosphyrus species (accounting for 14% of all *Anopheles* captured in Kampong Speu), *An. dirus A, Anopheles peditaeniatus, Anopheles nivipes, Anopheles tesselatus, Anopheles vagus, An. karwari, Anopheles kochi, An. sawadwongporni*, and seven other lesser captured species (Table 2).

**Table 2:** Anopheles species composition by timepoint and site

**#**	***Anopheles*** **Species (molecular)**	**Total**	Mondulkiri			Kampong Speu Village			Kampong Speu Forest		
			T0	T1	T2	T0	T1	T2	T0	T1	T2
1*	Subgroup Leucosphyrus	210	84	62	31	9	18	0	4	2	0
2	***An. aconitus***	4	0	0	0	1	2	0	0	1	0
3	*An. babirostris sl*	1	0	0	0	1	0	0	0	0	0
4	***An. dirus Form A***	199	51	14	92	3	19	16	1	2	1
5	*An. dirus sl*	25	2	11	7	0	5	0	0	0	0
6	*An. dissidens*	2	0	0	0	0	1	1	0	0	0
7	***An. karwari***	11	0	0	1	7	1	2	0	0	0
8	***An. kochi***	10	0	0	0	4	1	4	1	0	0
9	***An. maculatus***	34	31	0	3	0	0	0	0	0	0
10	***An. minimus***	1	0	0	0	0	0	1	0	0	0
11	***An. nivepes***	30	0	0	0	30	0	0	0	0	0
12	***An. peditaeniatus***	40	0	0	0	6	24	6	0	4	0
13	*An. rampae*	2	0	0	0	0	1	1	0	0	0
14	***An. sawadwongporni***	11	0	0	1	3	2	5	0	0	0
15	***An. sinensis***	1	0	0	0	1	0	0	0	0	0
16	*An. tesselatus*	16	0	0	0	1	8	7	0	0	0
17	***An. vagus***	11	0	0	0	11	0	0	0	0	0
–	*Failed identifications*	*42*	*10*	*7*	*13*	*2*	*3*	*4*	*2*	*0*	*1*
	Total	650	178	94	148	79	85	47	8	9	2

^*^Anopheles species #1 represents the novel species belonging to Subgroup Leucosphyrus. *Anopheles* species in bold are known malaria vectors

^*^ Represents a novel species belonging to the Subgroup Leucosphyrus

### Human landing rates (HLR) and human behaviour-adjusted HLR (HBO-adjusted HLR)

In Mondulkiri, Kampong Speu village, and Kampong Speu forest, *Anopheles* landing behaviour (HLR inside and outside sentinel sleeping structures (only outside for Kampong Speu forest structures)) were assessed by measuring genus-level *landing* rates (landings per person per night (lpn)) as a proxy for *biting* rates. Directly measured HLRs were then adjusted to account for human presence (inside, outside), time inhabitants went to sleep, and bed net use, that is, the HBO-adjusted HLR.

### Mondulkiri

**Fig. 1:**
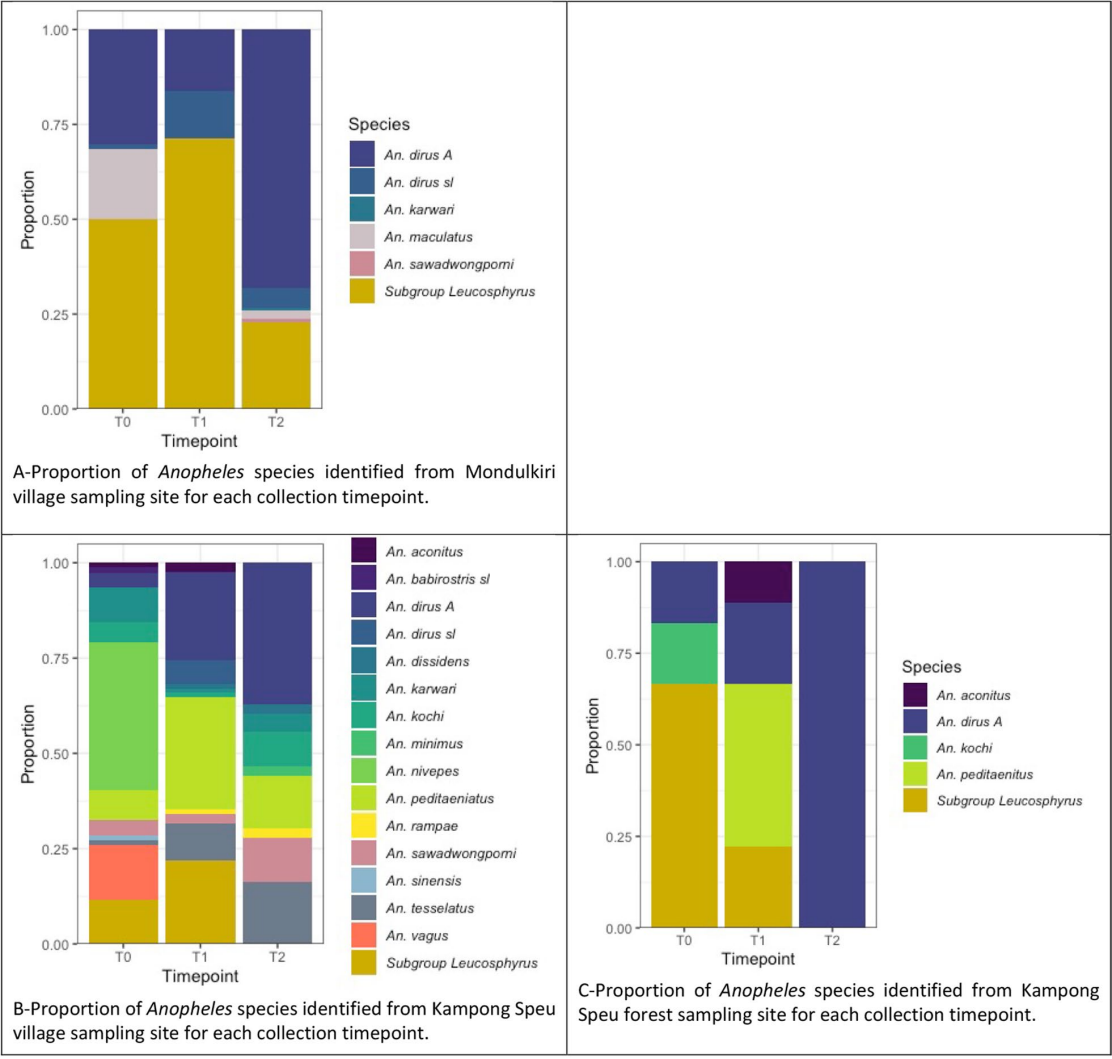
Proportion of Anopheles species by site and time point

The T0 HLR in Mondulkiri ranged from 0 to 0.4 lpn indoors (nightly mean 0.1 lpn) and from 0 – 1.48 lpn outdoors (nightly mean 0.4 lpn) (Table 2, Fig. 1). Peak landing time was between 22h00 and 23h00 for both inside and outside, and very few or no mosquitoes were captured anywhere between 06h00 and 18h00. T1 landing was observed throughout the night, with a higher HLR outdoors compared to indoors. The HLR ranged from 0 to 0.17 lpn indoors (nightly mean 0.05 lpn) and from 0.08 to 2.33 lpn outdoors (nightly mean 1.17 lpn) (Table 2, Fig. 1). Peak landing time outside was between 21h00 and 22h00, with the peak inside landing one hour later from 22h00 to 23h00. T2 Landing rates ranged from 0 and 0.04 lpn inside (nightly mean 0.00 lpn) and from 0.00 to 2.38 lpn outside (nightly mean 0.22 lpn). Mosquito landing was observed throughout the night during T2, with indoor landing only recorded between 19h00 and 22h00 (Table 2, Fig. 1), and most outside landing occurred between 18h00 and 19h00.

When integrating HLC data with HBO data, producing the HBO-adjusted HLR (Table 3), the primary human-vector exposure space for all timepoints during daytime hours (06h00–18h00) was when individuals were inside, awake, without using a bed net. During evening and early morning hours (18h00–06h00), the primary exposure space was when individuals were outside, awake, without a bed net.

**Table 3:** Human landing rate (lpn) by site and timepoint

	HLR range inside (lpn)	Nightly HLR mean inside (lpn)	HLR range outside (lpn)	Nightly HLR mean outside (lpn)
MDK village				
T0	0.00–0.40	0.1	0.00–1.48	0.4
T1	0.00–0.17	0.05	0.08–2.33	1.17
T2	0.00–0.04	0	0.00–2.38	0.22
KS village				
T0	0.00–0.38	0.07	0.00–1.48	0.1
T1	0.03–0.22	0.1	0.00–0.31	0.13
T2	0.06–0.28	0.13	0.03–0.14	0.05
KS forest				
T0	–	–	0.00–0.17	0.06
T1	–	–	0.08–1.17	0.57
T2	–	–	0.17–1.00	0.53

### Kampong Speu

**Fig. 2:**
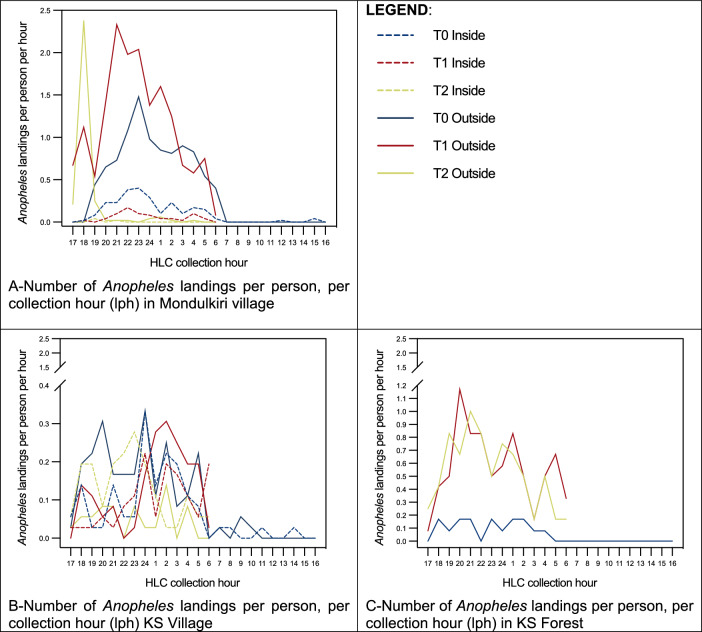
Number of Anopheles landings per person, per collection hour (lph) (dashed lines: outside landing; solid lines: inside landing; blue: T0; red: T1; yellow: T2)

**Fig. 3:**
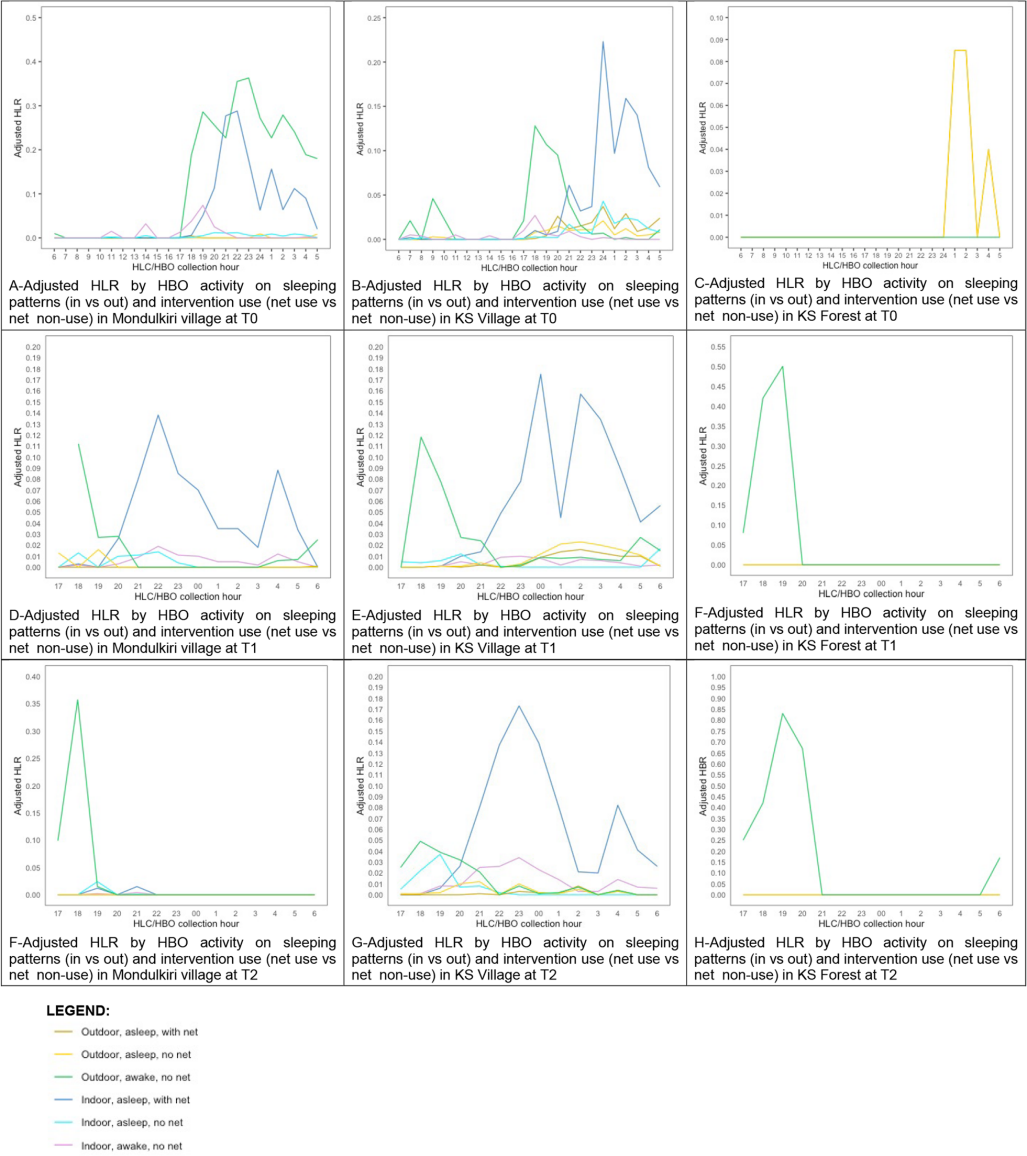
Adjusted HLR by HBO activity on sleeping patterns (in vs out) and intervention use (net use vs net non-use) in Mondulkiri village (**A**), Kampong Speu village (**B**), and Kampong Speu forest (**C**), across all three collection timepoints (T1, T2, T3)

There were two distinct sampling sites in Kampong Speu: a village site and a forest site. The T0 HLR in Kampong Speu village ranged from 0 to 0.38 lpn indoors (nightly mean 0.07 lpn) and from 0 to 1.48 lpn outdoors (nightly mean 0.1) (Table 2, Fig. 3). Peak HLR in the village was between 00h00 and 01h00 for both inside and outside. In the forest sites (where only outdoor data was collected), most mosquito captures occurred between 18h00 and 06h00, ranging from 0 to 0.17 lpn landings per hour (nightly mean 0.06 lpn) (Table 2, Fig. 3). In both village and forest settings, landing was observed throughout the night at T1 (Table 2). Outside HLRs in both the village (range: 0 and 0.031 lpn, nightly mean: 0.13 lpn) (Fig. 2) and the forest (range: 0.08–1.17 lpn, nightly mean: 0.57 lpn) (Fig. 3) were higher than inside village HLRs (range: 0.03–0.22 lpn, nightly mean: 0.1 lpn), and overall, outside HLRs in the forest were higher than HLRs in the village. During T2, landing was observed throughout the night in both the village and the forest sites. In the village, inside HLR was slightly higher than outside HLR (Table 2 and Fig. 2). Landing rates in the village ranged from 0.06 to 0.28 lpn inside (nightly mean 0.13 lpn) and from 0.03 to 0.14 lpn outside (nightly mean 0.05 lpn), while HLRs in the forest were higher, ranging from 0.17 to 1.00 lpn (nightly mean 0.53 lpn) (Fig. 3).

The HBO-adjusted HLR data (Table 3) suggests that during daytime and early evening hours (7h00–22h00), the primary human-vector exposure space was when individuals were outside, awake, without a bed net. As people went to bed at night, the primary exposure was for those people who were indoors, asleep, without a bed net (23h00–06h00). The only *Anopheles* exposure space at the forest sites was between 01h00 and 05h00 for people outdoors, awake, without a bed net. Towards the early morning in the village, outside and awake becomes the primary gap in protection again. In the forest, the primary gap in protection observed was in the early evening for those awake, outside (Table 4).

**Table 4:**
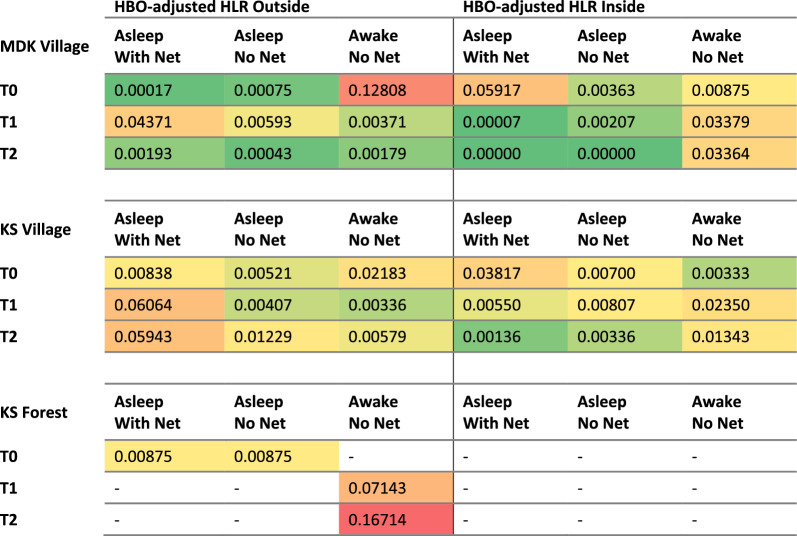
Mean adjusted human landing rate

Colour gradients represent 10% intervals of the total HBO-adjusted human landing rate range, ranging from light green (lowest 10%) to dark red (highest 10%)

### Observed or reported Project BITE tool use

#### Topical repellent

In Mondulkiri, during the topical repellent use observation hours (17h00–21h00 and 06h00–07h00), 12% reported using the topical repellent both indoors and outdoors during T1, with 4% and 1% reported during T2, respectively. Topical repellent use in Kampong Speu village sites was lower than in Mondulkiri, with 3% of individuals reporting using the repellent indoors and 10% reporting using the repellent outdoors during T1, with 0% indoor use and 4% outdoor use reported during T2. In Kampong Speu forest sites, no one reported using topical repellents, though it should be noted that few people were observed at this outdoor sampling site during the observation period (both T1 and T2), compared to Kampong Speu and Mondulkiri village sites and Mondulkiri sites.

#### Etofenprox-treated clothing

In Mondulkiri, relatively few people reported wearing etofenprox-treated clothing during the observation hours (17h00-21h00 and 06h00-07h00), with 2% reporting wearing treated clothing indoors and 7% outdoors during T1, with 9% indoors and 14% outdoors during T2. Between 17h00 and 21h00, the proportion of people wearing treated clothing decreased, both indoors and outdoors, as people prepared for sleep. Treated clothing use in Kampong Speu villages sites was lower than in Mondulkiri, with < 1% of individuals reported wearing treated clothing indoors and 2% outdoors during T1, and no people reporting wearing the treated clothing during T2 (either in the village or the forest sites).

#### BiteBarrier VPSR

Use of BiteBarrier was observed in all structures in both Mondulkiri and Kampong Speu, during both T1 and T2, and were generally not moved once hung. On delivery of the forest packs, households were instructed to hang at least two units of the BiteBarrier at a time. Over the 14 observation hours per structure (17h00–07h00), across six observation nights, there was an average of three units of BiteBarrier hung in structures in Mondulkiri during T1 and 3.5 units during T2. In Kampong Speu village sites, an average of 1.8 units were observed per structure during both T1 and T2 observations.

## Discussion

This study highlights the importance of identifying where and when people are exposed to *Anopheles* bites based on both *Anopheles* landing behaviour and human behaviour including sleep and wake patterns, as well as intervention use. Identifying gaps in protection is necessary to gain insight into how both present and novel mosquito bite prevention interventions can be targeted and evaluated for optimized vector control strategies.

### Anopheles* species diversity*

From a total of 17 *Anopheles* species across all three sampling sites, six species were found in Mondulkiri village, five species in Kampong Speu forest, and 16 in Kampong Speu village (Table 2). The same species were found across all three sites, with the exception of *An. vagus* found only in Kampong Speu village. Of these 17 *Anopheles* species, 11 are confirmed vectors of *Plasmodium* (Table 2) [37–43]. Species *An. dirus*, *An. maculatus*, and *An. minimus* comprise Cambodia’s major malaria vectors [44–46], and all but one species (Subgroup *Leucosphyrus*) have previously been described in Cambodia [15, 39, 40]. The predominant species collected in Mondulkiri during this study was a yet to be identified species belonging to the Subgroup Leucosphyrus, and which has not been reported elsewhere. This unknown species was also identified in Kampong Speu (Fig. 1). This study cannot determine whether this novel species could contribute to sustained transmission in Cambodia, as specimens were not screened for *Plasmodium*. Its predominance in Mondulkiri and elevated presence in Kampong Speu are noteworthy, and we recommend that further investigations be conducted to better understand the bionomics and potential role in transmission of this newly detected species. Due to the small subsample of *Anopheles* confirmed to species-level, species-specific biting trends (location, time) also cannot be inferred. Evidence of species compositions and their related bionomic traits is important when looking at what interventions might be most effective as well as how these interventions impact each species – differentially impacting transmission over time [47, 48].

### Human-mosquito exposure profiles (gaps in protection)

**Fig. 4:**
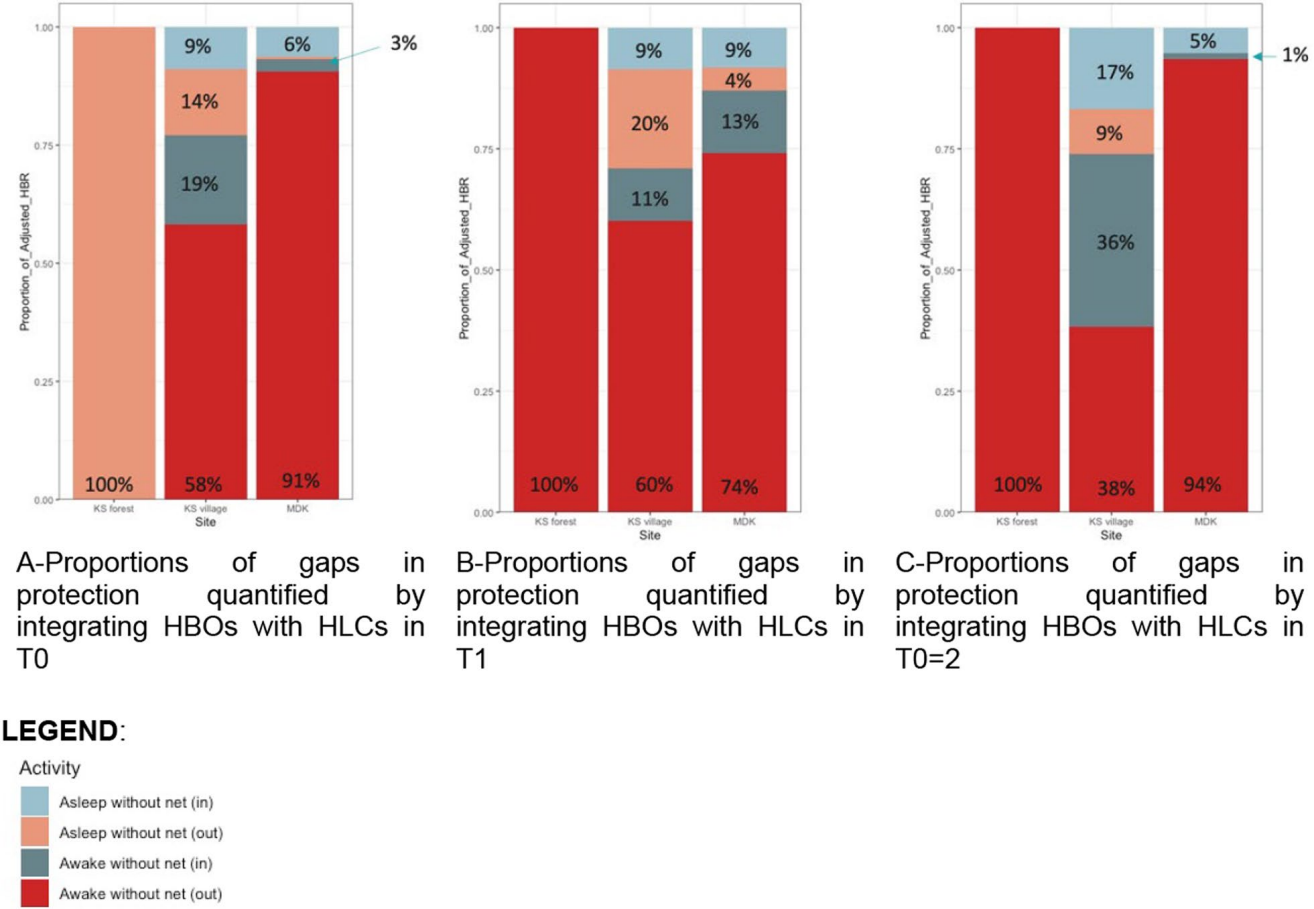
Proportions of gaps in protection quantified by integrating HBOs with HLCs

Across all three collection sites, human-*Anopheles* exposure was generally greatest when people were awake, outdoors, without the protection of a bed net, especially during the early evening hours. This finding highlights the relevance of including HBOs alongside HLCs to better identify human-vector exposure points, as the HLR results on their own would suggest elevated biting pressure (and thus presumed peak exposure) during the later evening hours and into the night (Fig. 2). However, as bed net use was common in the village setting, inhabitants were generally asleep under their nets during the later evening and nighttime hours, thereby resulting in the largest proportion of human-vector exposure during the earlier evening hours, when individuals were spending time outside the protection of their nets. The second most common gap in protection was when people were sleeping indoors without a net. During the early evening period outdoors, people were often socializing, cooking, or performing household tasks before going inside to prepare for sleep, and therefore bed nets, though widely available, offer no practical protection. Once inside in the later evening, most people from this study population were protected by bed nets as they went to sleep (Figs. 3, 4). Household members attempt to combat these bites by burning mosquito coils or leaves, boiling water, or using other types of techniques to stave off mosquitoes [22]. These behaviours suggest that people are aware that existing vector control tools are not adequate for complete protection and are willing to use additional methods to fill gaps in existing forms of bite protection.

### Insights on use of project BITE forest pack interventions

HBOs also included observations of the use of Project BITE tools at T1 and T2 timepoints. BITE tool use varied between Mondulkiri and Kampong Speu, with generally higher uptake observed in Mondulkiri. Topical repellent use was more common in Mondulkiri, particularly during T1, while Kampong Speu had lower reported use, especially in forest sites where no usage was recorded. Similarly, etofenprox-treated clothing was worn by more individuals in Mondulkiri than in Kampong Speu, where its use was minimal and absent by T2. In contrast, observed BiteBarrier VPSR use was more consistent across both locations, though Mondulkiri households tended to hang more units per structure than those in Kampong Speu. These patterns suggest that intervention uptake may be influenced by granular, local behavioural differences and site-specific factors, such as daily activity patterns and environmental contexts [49–51].

In addition to non-use, the study team also observed incorrect use of both the BiteBarrier and topical repellent. For example, some BiteBarrier units were observed hung in a rolled-up fashion, rather than hanging free like a flat sheet of paper, or hung too low (ideal hanging height is around 1.5 m off the ground), and either too many or too few units hung in a given area (SBCC materials indicated that two units of BiteBarrier were to be hung in a room, though the volume of that room was not specified). For the topical repellent, which is designed to be applied to exposed skin, people were observed (or reported) to have sprayed the repellent directly on insects (in the fashion of an aerosol insecticide) or sprayed in the air in areas like toilets. Low use of tools may also be the result of dislike or disapproval of the tools, or that the tools were not appropriate for peoples’ lifestyles or habits. At the same time, individuals were encouraged to use tools in the forest where malaria risk is highest, which could potentially explain some low or non-use in village settings. While the present study was not designed to assess acceptability or appropriateness of the tools, other studies within Project BITE are investigating this. These observations offer insight about the importance of sustained community engagement and fit-for-purpose SBCC to optimize adherence and correct product usage [22]. As has been documented previously with both VPSRs and topical repellents [50, 52], the availability of an intervention does not guarantee it’s appropriate use; and even if an appropriate tool is provided to close a gap in protection, it is of no use if the tool is not used in the appropriate manner.

### Study limitations

While earlier studies have used the combined HLC and HBO methodology to evaluate LLIN use [35, 36, 53], this is the first study in which HBOs and HLCs have been used to evaluate gaps in protection when considering mosquito bite prevention interventions other than bed nets. Some key challenges were observed. First, while each of the living structures included in the HLC and HBO activities had received access to the BITE tools, staff performing the data collection were not aware of whether individuals being counted during HBOs were a part of the observed household or not. For instance, an individual who is recorded as not using topical repellent inside a living structure may not be “officially” part of that household (i.e., might be a guest, a family member from another household), and therefore may not have received the topical repellent. As a result, the proportion of individuals observed using the BITE tools does not consider whether that particular individual had access to the tools.

Second, this study focused on understanding gaps in protection at the village or household level. Two forest sentinel sampling structures were constructed by the study team in Kampong Speu, but these were located at the forest fringe. For reasons primarily concerning staff safety, collections and observations in the deep forest were not possible. Therefore, results concerning existing gaps in protection from this study should not be extended, without further research, into true deep forest settings. Gaps in protection observed in a village setting could be different from those observed in a forest setting, as mosquito biting behaviours [16, 17] and human behaviours around intervention use and activities may differ [8]. Still, the implementation of “environment agnostic” tools (those which can be used no matter the wind or rain conditions), such as topical repellents and treated clothing, are likely well-suited to improve personal bite protection in forest settings [21, 54–56]. In fact, even though the World Health Organization (WHO) currently refrains from establishing formal recommendations on ITCs and topical repellents applications, it does suggest these interventions for personal protection and considers their utilization for high-risk groups who are not protected by other bite protection interventions [57]. Project BITE tools were demonstrated to reduce mosquito landing in a field study when used in combination [21], and semi-field system studies have found that they are also each effective when used individually.

### Towards supporting Cambodia’s malaria elimination goals

In 2022, there were a total of 4,053 confirmed malaria cases (all species), with a 66% reduction (1,384) in confirmed cases in 2023 [14]. Of those total cases in 2023, only 2.5% (34) were *P. falciparum* cases, compared to 9.8% of all cases in 2022, indicating a substantial reduction in remaining transmission. This trend reflects the commitment of the Cambodia national malaria programme, provincial health departments, and other health authorities to reaching their target of the elimination of all malaria species in Cambodia by 2025 [2].

Across all collection sites and timepoints in this study, outdoor *Anopheles* biting constituted the predominant gap in protection, pointing to the need for additional, bite prevention intervention strategies which supplement—but do not necessarily replace—existing vector and bite protection tools. Preceding results from Project BITE on the efficacy of tools in the BITE forest pack (VPSR, insecticide treated clothing, and topical repellent) against wild, local *Anopheles* landing [21] and on key secondary life history traits suggest that these tools have the potential to help further reduce forest-based *Anopheles* exposure in Cambodia. Furthermore, Project BITE and other modeling assessments [21, 58] demonstrated that these or similar [59] spatial repellent interventions reduced vectoral capacity, suggesting potential for community-level impact, and corroborate findings from other recent semi-field [60–65] and field studies [66–68] of VSPRs. Owing to this expanding body of evidence, insecticidal personal protection interventions are increasingly recognized as promising for public health use, though additional evidence of epidemiological impact is needed for WHO to establish a policy recommendation for these interventions [24].

To reach complete malaria elimination in Cambodia and across the GMS, populations—especially high-risk, vulnerable, and mobile groups—must be provided with safe, effective, and acceptable tools which supplement existing methods of bite protection. Research should prioritize developing, improving, and providing those tools which best help close the current gaps in protection, which should be tailored to locally specific contexts. Additionally, to successfully implement and sustain novel, repellent interventions, it is crucial to identify factors that influence their acceptance and use, such as risk perception [22], past exposure to vector control, and cost [49, 50]. A study in Mondulkiri Province found that while VPSRs were generally accepted, indoor use could be limited by a preference for bed nets, whereas outdoor use in the peridomestic area could be more effective. The study highlights the need for locally available products and further research to understand how cultural and social factors affect the adoption of these tools [50]. Given the diversity in population groups in Cambodia (and in other Southeast Asian countries), additional social studies are required to better understand how the interplay between human behaviour shaped by varying cultural and social contexts and vector behaviour might lead to, or hinder, successful implementation of novel bite prevention tools [51].

## Conclusion

This study demonstrates the importance of understanding where and when exposure to mosquito bites occur and where gaps in protection exist in two provinces in Cambodia. Although bed nets were available at all sites included in the study, they can only provide protection to people for a portion of time when people are potentially exposed. New tools are needed to fill the identified gaps in protection—most notably when people are outdoors in the early evening. The novel tools provided to the populations through Project BITE (VPSRs, topical repellent, and insecticide-treated clothing) demonstrate high entomological efficacy, but were observed to be underutilized and often misused, underscoring the need for robust community engagement and SBCC with any new intervention. This study confirmed high vector species diversity at study sites and identified a novel species that should be further investigated as a potential malaria vector with potential implications for malaria elimination. Cambodia is on the precipice of malaria elimination and must continue its innovative, adaptive, and aggressive approach with both existing and novel tools to ultimately reduce cases to zero.

## Data Availability

All data generated or analysed during this study are included in this published article and are available upon request.

## Abbreviations

BITE: Bite interruption toward elimination
CNM: Cambodia's National Center for Parasitology, Entomology, and Malaria Control
GMS: Greater Mekong subregion
HBO: Human behaviour observation
HLC: Human landing catch
HLR: Human landing rate
ITC: Insecticide treated clothing
LLIHN: Long-lasting insecticide treated hammock net
LLIN: Long-lasting insecticidal net
LPH: Landings per hour
RDT: Rapid diagnostic test
SBCC: Social and behavioural change communication
TR: Topical repellent
VPSR: Volatile pyrethroid spatial repellent
